# Both Neurons and Astrocytes Exhibited Tetrodotoxin-Resistant Metabotropic Glutamate Receptor-Dependent Spontaneous Slow Ca^2+^ Oscillations in Striatum

**DOI:** 10.1371/journal.pone.0085351

**Published:** 2014-01-15

**Authors:** Atsushi Tamura, Naohiro Yamada, Yuichi Yaguchi, Yoshio Machida, Issei Mori, Makoto Osanai

**Affiliations:** 1 Department of Radiological Imaging and Informatics, Tohoku University Graduate School of Medicine, Sendai, Japan; 2 Core Research for Evolutional Science and Technology, Japan Science and Technology Agency, Kawaguchi, Japan; 3 Division of Electrical, Electronic and Information Engineering, Graduate School of Engineering, Osaka University, Suita, Japan; 4 Department of Medical Imaging and Applied Radiology, Tohoku University Graduate School of Medicine, Sendai, Japan; University of California, Merced, United States of America

## Abstract

The striatum plays an important role in linking cortical activity to basal ganglia outputs. Group I metabotropic glutamate receptors (mGluRs) are densely expressed in the medium spiny projection neurons and may be a therapeutic target for Parkinson's disease. The group I mGluRs are known to modulate the intracellular Ca^2+^ signaling. To characterize Ca^2+^ signaling in striatal cells, spontaneous cytoplasmic Ca^2+^ transients were examined in acute slice preparations from transgenic mice expressing green fluorescent protein (GFP) in the astrocytes. In both the GFP-negative cells (putative-neurons) and astrocytes of the striatum, spontaneous slow and long-lasting intracellular Ca^2+^ transients (referred to as slow Ca^2+^ oscillations), which lasted up to approximately 200 s, were found. Neither the inhibition of action potentials nor ionotropic glutamate receptors blocked the slow Ca^2+^ oscillation. Depletion of the intracellular Ca^2+^ store and the blockade of inositol 1,4,5-trisphosphate receptors greatly reduced the transient rate of the slow Ca^2+^ oscillation, and the application of an antagonist against mGluR5 also blocked the slow Ca^2+^ oscillation in both putative-neurons and astrocytes. Thus, the mGluR5-inositol 1,4,5-trisphosphate signal cascade is the primary contributor to the slow Ca^2+^ oscillation in both putative-neurons and astrocytes. The slow Ca^2+^ oscillation features multicellular synchrony, and both putative-neurons and astrocytes participate in the synchronous activity. Therefore, the mGluR5-dependent slow Ca^2+^ oscillation may involve in the neuron-glia interaction in the striatum.

## Introduction

The calcium ion (Ca^2+^) is an important messenger for signal transduction, and intracellular Ca^2+^ concentrations ([Ca^2+^]_i_) change in response to various physiological stimuli in both excitable and non-excitable cells [1]–[3]. Ca^2+^ is a universal and versatile signal transduction molecule [1]. Intracellular Ca^2+^ can modulate the functions of proteins such as enzymes and receptors, gene expression, and morphological changes in cellular processes. The endoplasmic reticular (ER) Ca^2+^ store is a source of [Ca^2+^]_i_ elevation and is crucial for second messenger-induced intracellular Ca^2+^ signaling [4], [5]. Therefore, the Ca^2+^ released from the ER should contribute to the modulation of neuronal signal processing in the central nervous system.

In the basal ganglia, the striatum receives inputs from the cortex and is thought to play a crucial role in controlling somatic motor movements, behavioral patterns, cognition, learning, and memory [6], [7]. There are many types of metabotropic receptors that may contribute to intracellular Ca^2+^ signaling in the striatum [8]–[10]. Metabotropic glutamate receptors (mGluRs) are one class of candidate modulators for intracellular Ca^2+^ signaling in the striatum [8]. Striatal neurons and astrocytes express the abundant mGluR type 5 (mGluR5) [11]–[13], which is known to couple to phospholipase C (PLC) and to generate inositol 1,4,5-trisphosphate (IP_3_). mGluR5 has also been suggested as a therapeutic target for Parkinson's disease [14]–[16] and may interact with dopamine signaling via Ca^2+^ [10]. Dopamine signaling is essential for neuronal functioning in the striatum. Thus, mGluR5 is expected to play a role in information processing in the striatum.

We previously reported long-lasting spontaneous [Ca^2+^]_i_ oscillations in the rat striatum [17], which lasted up to about 250 s. These Ca^2+^ oscillations were not induced by action potentials, but induced by Ca^2+^ release from ER. In this previous study, we did not determine whether the spontaneous [Ca^2+^]_i_ transients occurred in neurons or astrocytes, nor did we identify the induction mechanism of the Ca^2+^release from ER. Therefore, in this paper, we identified the cell types exhibiting the spontaneous [Ca^2+^]_i_ transients using transgenic mice expressing green fluorescent protein (GFP) in astrocytes, and determine one of the induction mechanisms. We also analyze the cellular correlations of the slow Ca^2+^ oscillations. The preliminary results of this work have been previously reported in an abstract form [18].

## Materials and Methods

### Ethics statement

The Tohoku University Committee for Animal Experiments (Permit Number: 2010MdA-287, 2011MdA-292, 2012MdA-252, 2013MdA-343) and the Institutional Animal Care and Use Committee of the Graduate School of Engineering at Osaka University (Permit Number: 17-6-0) approved all animal experiments, and the experiments were performed in accordance with the Guidelines for Animal Experiments and Related Activities of Tohoku University and Osaka University, as well as the guiding principles of the Physiological Society of Japan and the National Institutes of Health (NIH), USA.

### Slice preparation

Transgenic mice expressing GFP under the control of the astrocyte-specific glial fibrillary acidic protein (GFAP) promoter were purchased from Jackson Laboratories (FVB/N-Tg(GFAPGFP)14Mes; Bar Harbor, ME) [19], [20]. The colony was maintained by crossing with C57BL/6, and mice crossed with C57BL/6 more than three times were referred to as GFAP-GFP mice. Corticostriatal slice preparations were performed as previously described [17], [21], [22]. Briefly, postnatal day 10 (P10) to P28 GFAP-GFP mice of either sex were anesthetized with halothane and decapitated. The cerebrum was rapidly isolated and placed in ice-cold artificial-cerebrospinal fluid (ACSF) bubbled with 95% O_2_-5% CO_2_. The composition of ACSF was as follows (in mM): 137 NaCl, 2.5 KCl, 0.58 NaH_2_PO_4_, 1.2 MgCl_2_, 2.5 CaCl_2_, 21 NaHCO_3_, and 10 glucose. Corticostriatal sagittal slices (300 µm thick) were prepared using a vibratome tissue slicer (VT-1000S or VT-1200S, Leica Microsystems, Wetzlar, Germany). The slices were incubated at room temperature in a submerged chamber containing gassed ACSF for at least 60 min prior to the experiments.

### Ca^2+^ imaging

[Ca^2+^]_i_ in the slices was measured using the membrane-permeant acetoxymethyl (AM) ester of Fura-2 LR (Fura-2 LR/AM, Calbiochem, San Diego, CA; [23]) dissolved in dimethylsulfoxide (DMSO; Dojindo Laboratories, Kumamoto, Japan). The dye-loading methods used were as previously described [17], [21], [22], [24]. In brief, the corticostriatal slice was placed in a small plastic Petri dish containing 100 µl ACSF with 20 µM Fura-2 LR/AM and 0.02% Cremophor EL (Sigma, St. Louis, MO). The dish was incubated at 35°C for 40 min in a small chamber, which was humidified and continuously aerated with 95% O_2_-5% CO_2_, and then washed with 100 µl ACSF at 35°C for 15 min. Fura-2 LR-loaded slices were transferred to a continuously superfused (2–2.5 ml/min) chamber on the stage of an epifluorescent upright microscope (BX51WI, Olympus, Tokyo, Japan). [Ca^2+^]_i_ changes were imaged with a 20×, NA 0.95 water-immersion objective (Olympus). The Fura-2 LR-loaded slices were alternately excited at wavelengths of 340 and 380 nm using a filter changer (Lambda DG-4, Sutter Instruments, Novato, CA; exposure time of 50–100 ms for each individual wavelength), and fluorescent signals were captured (F340 and F380) every 2 s with a cooled CCD (Cool SNAP HQ, Photometrics, Tucson, AZ) or an EM-CCD (DU-885, Andor, Belfast, UK). All equipment was controlled by MetaFluor software (Molecular Devices, Downingtown, PA). The experiments were performed under temperature control (30±1°C).

### Data analysis

Image analysis was performed with MetaFluor and custom-made programs written in MATLAB (Math Works, Natick, MA). In the measurement of Ca^2+^ signals from imaged cells, we identified Fura-2 LR-loaded cells in images of the slices and measured the average fluorescence (F340 and F380) within the region of interest (ROI) of these cells as a function of time. To avoid measurement of [Ca^2+^]_i_ in a fraction of the glial cells or vessel related cells, ROI was put only in the somatic region with round shape. [Ca^2+^]_i_ in a striatal cell was estimated by the fluorescence ratio (R  =  F340/F380) from each imaged cell [25]. To reduce the noise, we applied the Hanning filter (window length: 6 s) as a low-pass filter. The onset of each [Ca^2+^]_i_ transient for every cell was determined using an algorithm that defined the onset as the frame after which the slope of R was larger than a given set threshold. Then, the baseline was set to the mean R value of three frames before the onset, and the change in the R value from the baseline was defined as ΔR. To eliminate false positives, [Ca^2+^]_i_ transients with peaks that did not exceed the threshold (ΔR  = 0.005–0.01) were discarded. Some remaining false positives were deleted upon visual inspection. When we showed the traces of the time courses of R, the baselines were subtracted from the raw R trace. The baseline of each trace was obtained by spline interpolating the local minimum values of the trace using a higher order Hanning filter (window length, 34–54 s) [17].

To determine the properties of individual [Ca^2+^]_i_ transients included in the slow Ca^2+^ oscillations, we calculated the transient rate, amplitude, duration, rise slope, and decay slope. The transient rate was defined as the number of onsets per unit time during recording period (>1000 s). The duration indicates the time from onset until the decay to threshold. The rise and decay slopes are the slopes of a regression line for the data points in 5–90% of the peak during the rise phase and in 95% to 1/e of the peak during the decay phase of the [Ca^2+^]_i_ transients, respectively.

### Statistical analysis

All data are presented as means ± s. e. m. (standard error of the mean), and differences were considered significant at p<0.05 by statistical testing, unless stated otherwise. The statistical significance was assessed based on Mann-Whitney U test or Wilcoxon signed-rank test for the comparison between the mean values of unpaired two groups or paired two groups, respectively, or Kruskal-Wallis test with Steel-Dwass post-hoc test for the multiple comparisons. One sample t-test was used to demonstrate the significance of the effect of the pharmacological reagent compared with the control condition. Kolmogorov-Smirnov test was used for the comparison between the distributions of the two groups. P-values are two-sided. The law of propagation errors and the method of interval estimation were applied for the estimation of the probabilities in the immunohistochemistry.

### Multicellular correlation analysis of the slow Ca^2+^ oscillations

The evaluation of multicellular correlations in the slow Ca^2+^ oscillations was performed by the following method based on previous reports [26]–[28]. The time series data for R in individual cells were binarized by the threshold described above. To determine whether the slow Ca^2+^ oscillations recorded from different cells were correlated, the numbers of simultaneous activations that included more cells than expected by chance were detected. To do this, Monte Carlo simulations with 1000 replications were used to estimate the significance of their multicellular combined high-Ca^2+^ state. The threshold corresponded to a significance level of p<0.01. Because the Ca^2+^ transients we analyzed had long durations, we estimated the correlation level for the summation of the time spent in the combined high-Ca^2+^ state.

### Immunohistochemistry

The brains of transgenic and non-transgenic mice were fixed with 4% paraformaldehyde in PBS (pH 7.4) for 24 hr at 4°C. Vibratome sections (40 µm thick) of brain regions were cut in cold PBS, transferred to slides, and incubated for 4–12 hr at 4°C in PBS containing 0.1% (v/v) Triton X-100 and 10% nonimmune goat serum. Sections were then washed for 3×15 min in PBS. Sections from transgenic mice were incubated for 24 hr at 4°C with a rat anti-GFP monoclonal antibody (Nacalai Tesque, Kyoto, Japan) and either a rabbit anti-bovine S-100 polyclonal antibody (Sigma) or a mouse anti-NeuN monoclonal antibody (Chemicon International, Temecula, CA) at 1∶1000 dilutions in PBS containing 0.01% Triton X-100 and 1% nonimmune goat serum. The sections were again washed for 3×15 min in PBS and then incubated 4–8 hr at 4°C with a fluorescein-conjugated goat anti-rat IgG antibody (Chemicon International) and either rhodamine-conjugated goat anti-rabbit IgG antibody (Sigma) or rhodamine-conjugated goat anti-mouse IgG antibody (Chemicon International) at a 1∶50 dilution in PBS containing 0.01% Triton X-100 and 1% nonimmune goat serum. The sections were then rinsed in four changes of PBS and coverslipped with an antifade reagent (ProLong Gold, Invitrogen, Carlsbad, CA). For sections from non-transgenic mice, the first antibody pair was substituted with the rabbit anti-bovine S-100 polyclonal antibody and the mouse anti NeuN monoclonal antibody, and the second antibody pair was substituted with the rhodamine-conjugated goat anti-rabbit IgG antibody and a fluorescein-conjugated goat anti-mouse IgG antibody (Chemicon International). The fluorescence of fluorescein and rhodamine was visualized in the same sections, using standard fluorescein and rhodamine filters, respectively, by epifluorescent microscopy (BX-51WI, Olympus). In the astrocytes, GFAP is not only expressed in soma but also in the cellular processes. On the other hand, S-100 is mainly expressed in the somatic region of the astrocytes [29]. To avoid counting the processes of the astrocytes, we used the anti-S100 antibody for labeling the glial cells instead of the GFAP antibody.

### Drugs

All drugs were applied by perfusion. Tetrodotoxin (TTX; 1 µM; Alomone Labs, Jerusalem, Israel) was used to block action potentials. 6-cyano-7-nitroquinoxaline-2, 3-dione (CNQX; 10 µM; Tocris, Bristol, UK) and DL-2-amino-5-phosphonovaleric acid (AP5; 50 µM; Tocris) were used to block AMPA-type and NMDA-type ionotropic glutamate receptors, respectively. Thapsigargin (2 µM; Alomone Labs) was used to deplete the intracellular Ca^2+^ store by blocking the Ca^2+^ ATPase of the Ca^2+^ store. 2-aminoethoxydiphenyl borate (2-APB; 100 µM; Tocris) was used to block the IP_3_ receptor, which is well known for releasing Ca^2+^ from the Ca^2+^ store. 2-methyl-6-(phenylethynyl)-pyridine (MPEP; 10 or 30 µM; Tocris) was used to block mGluR5. (+)-2-methyl-4-carboxyphenylglycine (LY367385; 50 µM; Sigma) was used to block mGluR1.

In the pharmacological experiments, we discarded any cells that did not show [Ca^2+^]_i_ transients within 600 s just before the application of pharmacological agents to confirm their effects.

## Results

### Both putative-neurons and astrocytes in the striatum exhibit slow Ca^2+^ oscillations

**Figure 1:**
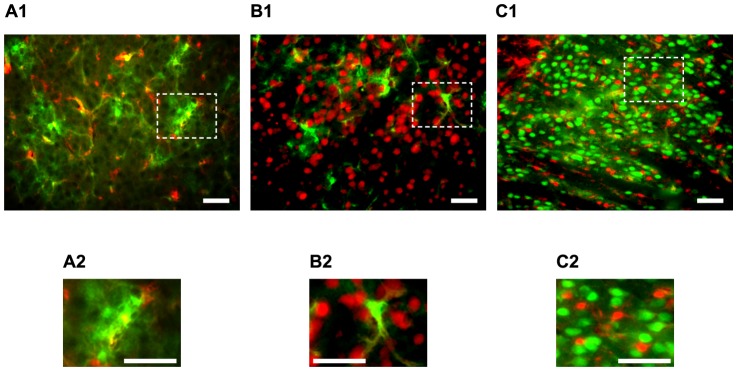
GFP was expressed only in astrocytes, and GFP-negative cells in GFAP-GFP mice were mainly neurons. A1, 2, Expression of GFP (green) and S-100 (red), a marker of astrocytes, in striatal slices of the GFAP-GFP mouse. Most S-100-positive cells expressed GFP. The proportions of cells positive for both GFP and S-100, for only S-100, and for only GFP were 62.4±1.8%, 30.3±1.9%, and 7.2±1.1%, respectively (n = 5 slices, total cell number  = 1509 cells). B1, 2, Expression of GFP (green) and NeuN (red), a marker of neurons. Few double-stained cells were observed (n = 4 slices). C1, 2, NeuN (green) and S-100 (red) expression in striatal slices of non-transgenic mice. In the striatum, fewer astrocytes were observed than neurons, and the proportion of NeuN-positive cells to S-100-positive cells was approximately 2∶1 (n = 3 slices). A2, B2, C2, The magnified images in the dashed boxes shown in A1, B1, C1, respectively. Scale bars, 50 µm.

To determine whether neurons or astrocytes exhibit the slow Ca^2+^ oscillation, we used GFAP-GFP mice. First, we confirmed that the GFP-positive cells were astrocytes and that the GFP-negative cells were neurons by immunohistochemistry (Figure 1). We used the anti-NeuN antibody as the neuronal marker, and the anti-S-100 antibody as the astrocytic marker (see Materials and Methods). The densities of cells positive for both GFP and S-100, for only S-100, and for only GFP were 426±120, 205±69 and 46±25, respectively (/mm^2^, n = 5 slices, 2 mice, total counted cell number  = 1509 cells, mean ± SD). Therefore, the proportion of GFP-negative cells to S-100-positive cells was 30.3±7.3% (mean ± SD). The densities of cells positive for S-100, and for NeuN, were 636±154 (n = 5 slices, 4 mice, total counted cell number  =  2004 cells), and 1538±298 (n = 5 slices, 4 mice, total counted cell number  = 1659 cells), respectively (/mm^2^, mean ± SD). Therefore, the proportions of S-100-positive cells to the all immunostained cells were 29.2±6.4% (mean ± SD). Hence, the probability of the GFP-negative cells being astrocytes (S-100-positive cells) was 8.9±2.9% (mean ± SD; propagation of errors) and 95% confidence interval of this probability was 8.9±5.7%. S-100 was also expressed in the oligodendrocytes [30]. There is a report that the S-100 was expressed in microglia [31]. Therefore, the probability that GFP-negative cells were glial cells was estimated to 3.2–14.6%, statistically (interval estimation). On the basis of this result and according to the previous report [19], in this paper, we treated GFP-positive cells as astrocytes, and GFP-negative cells as putative-neurons (see Discussion).

**Figure 2:**
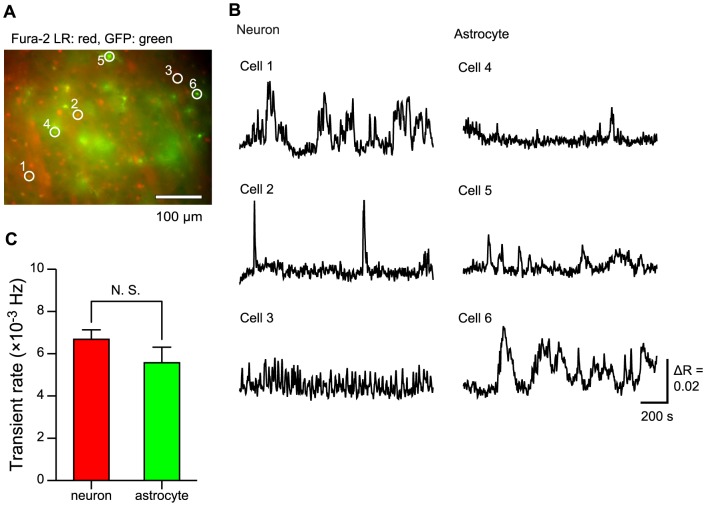
Slow Ca^2+^ oscillations in striatal cells. A, Fluorescence image of a striatal slice from a GFAP-GFP mouse. This image is an artificially colored merged fluorescence image of Fura-2 LR (red) and GFP (green). Cells 1–3 are putative-neurons because they are GFP-negative, and cells 4–6 are astrocytes because they are GFP-positive. Scale bar, 100 µm. B, Time courses of the slow Ca^2+^ oscillations. Cell numbers correspond to the region numbers in A. Scale bar, 200 s, ΔR  = 0.02. C, Comparison of the transient rate of the slow Ca^2+^ oscillations between putative-neurons and astrocytes. The number of cells recorded is 200 putative-neurons and 78 astrocytes. N. S.: no significant difference.

A fluorescence image of the Fura-2 LR-loaded cells in a striatal slice of GFAP-GFP mouse is shown in Figure 2A. GFP fluorescence was observed in cells 4–6, but not in cells 1–3. Therefore, the cells 1–3 were putative-neurons, and the cells 4–6 were astrocytes. On average, 13.3±1.8 putative-neurons and 5.2±0.6 astrocytes were found to exhibit the slow Ca^2+^ oscillations in a field of view (450 µm×330 µm or 333 µm×334 µm; n = 15 slices, 14 mice). Typical time courses of the slow Ca^2+^ oscillations are shown in Figure 2B. The sampling interval of the Ca^2+^ transients was long (2 s) (see Materials and Methods). Therefore, we ascribed all elevations of the Ca^2+^ more than the threshold level to the slow Ca^2+^ oscillations. For example, cell 1 exhibited bursts of Ca^2+^ transients and long-lasting Ca^2+^ transients. Cell 3 exhibited Ca^2+^ transients of short duration, high frequency, and small amplitude. Cell 4 exhibited Ca^2+^ transients of low frequency and small amplitude. Cell 6 repeatedly exhibited long-lasting Ca^2+^ transients. An individual cell exhibited a mixed profile of these patterns and we were not able to classify the cells in terms of these patterns. The slow Ca^2+^ oscillations exhibited various patterns, but they all had common features in manifesting spontaneity and repetition. The transient rates did not differ between putative-neurons and astrocytes (Figure 2C; (6.68±0.45) ×10^−3^ Hz in putative-neurons, (5.57±0.74) ×10^−3^ Hz in astrocytes, n = 200 putative-neurons and 78 astrocytes, 15 slices, 14 mice; p = 0.0903; Mann-Whitney U test).

**Figure 3:**
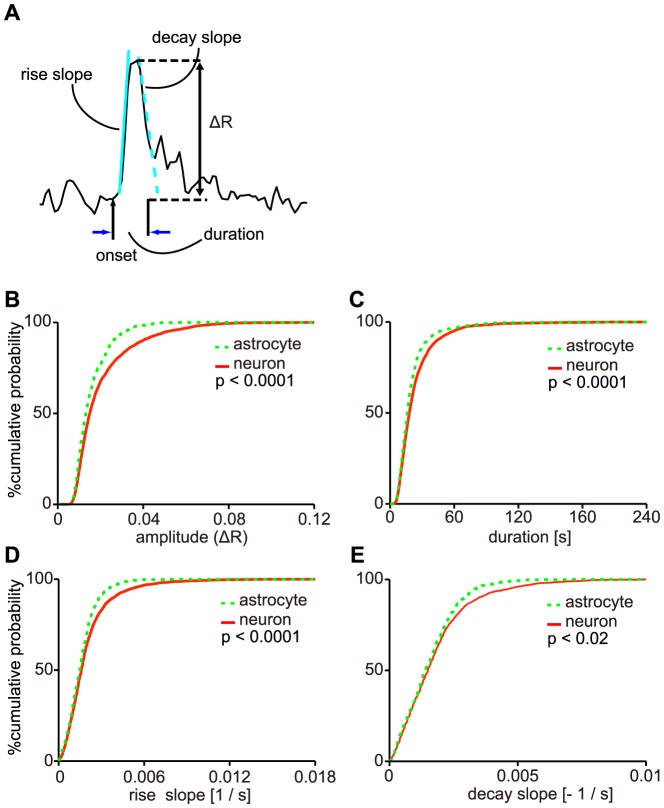
Differences in properties of the slow Ca^2+^ oscillations between striatal putative-neurons and astrocytes. A, Schematic illustration of four parameters of a Ca^2+^ transient. The peak amplitude (ΔR), duration, rise slope, and decay slope are indicated for each value in this figure. B–E, The distributions of the peak amplitude (B), duration (C), rise slope (D), and decay slope (E) of the Ca^2+^ oscillations in putative-neurons and astrocytes in cumulative probability plots. The solid line indicates the distribution of each parameter for the Ca^2+^ oscillations in putative-neurons, and the dashed line indicates the distribution of each parameter for the Ca^2+^ oscillations in astrocytes. *P*- values from the Kolmogorov-Smirnov test are shown in the plots.

To characterize the slow Ca^2+^ oscillations, individual events were dissolved from the entire R trace in a record, and four parameters, the peak amplitude, duration, rise slope, and decay slope, were extracted (Figure 3A). The distributions of those four parameters were shown in Figure 3B–E. The median values of the peak amplitude, duration, rise slope, and decay slope in putative-neurons were (1.50±1.43) ×10^−2^, 18±20 s, (1.60±1.70) ×10^−3^, and (1.50±1.42) ×10^−3^ (n = 3200 events, 189 cells, 21 slices, 20 mice, median ± standard deviation (SD)), and those values in astrocytes were (1.32±0.77) ×10^−2^, 18±16 s, (1.49±1.03) ×10^−3^, and (1.40±1.04) ×10^−3^ in astrocytes (n = 786 events, 71 cells, 21 slices, 20 mice, median ± SD), respectively. All of those four parameters were significantly different between putative-neurons and astrocytes (Fig. 3B–E; p<0.0001, p<0.0001, p<0.0001 and p = 0.0102, respectively; Kolmogorov-Smirnov test). These results indicated that the individual events of the Ca^2+^ oscillations in putative-neurons tended to have larger amplitude, longer duration, faster rise, and decay compared with those of astrocytes. The duration of the 5% events of the spontaneous Ca^2+^ oscillation was longer than a minute in putative-neurons. This is the first report of the Ca^2+^ oscillation, in which single events prolonged more than a minute, in striatal putative-neurons of acute slice preparations without any activation, under cell-type discrimination.

**Figure 4:**
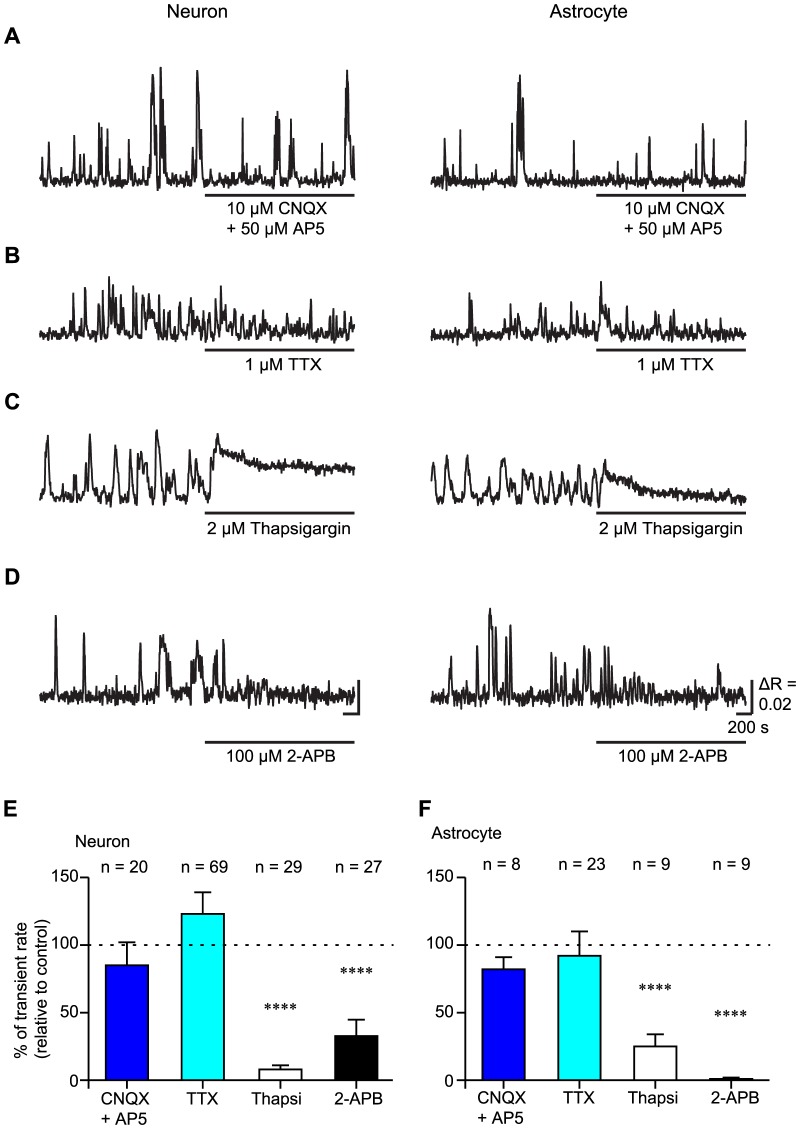
The slow Ca^2+^ oscillations in both putative-neurons and astrocytes were mainly due to Ca^2+^ release from the intracellular Ca^2+^ store via the IP_3_ receptor. A–D, Typical time courses of the slow Ca^2+^ oscillations during the administration of 10 µM CNQX and 50 µM AP5 (CNQX + AP5), 1 µM TTX, 2 µM thapsigargin (Thapsi), and 100 µM 2-APB in putative-neurons and astrocytes. Horizontal bars under the time courses indicate the application period of the agents. Scale bar, 200 s, µR  = 0.02. E, F, Transient rates of the slow Ca^2+^ oscillations during the administration of various pharmacological agents in putative-neurons (E) and astrocytes (F). The transient rates of the slow Ca^2+^ oscillations are normalized by the transient rates under control conditions. The number of cells recorded is shown above each bar graph. ****p<0.001; one-sample t-test.

To determine the induction mechanisms of the slow Ca^2+^ oscillations, pharmacological experiments were conducted (Figure 4). The slow Ca^2+^ oscillations were not blocked by the application of 10 µM CNQX and 50 µM AP5 or 1 µM TTX (Figure 4A, B), indicating that the slow Ca^2+^ oscillations in both putative-neurons and astrocytes were not induced by excitatory synaptic transmission or action potentials (Figure 4E, F; transient rate relative to the control: 102±17% (n = 20 putative-neurons, 4 slices, 4 mice; p = 0.3886) and 91±9% (n = 8 astrocytes, 3 slices, 3 mice; p = 0.0856) with CNQX and AP5 administration, and 123±16% (n = 69 putative-neurons, 4 slices, 4 mice; p = 0.1391) and 92±18% (n = 23 astrocytes, 4 slices, 4 mice; p = 0.6471) with TTX administration; one-sample t-test). Although average of the transient rates of the slow Ca^2+^ oscillations were not changed by blockade of excitatory synaptic transmissions or action potentials significantly, TTX administration altered other parameters (amplitude, duration, rise slope and decay slope) of the slow Ca^2+^ oscillations in putative-neurons as described in a later section. To determine the contribution of the Ca^2+^ release from the intracellular Ca^2+^ store to the slow Ca^2+^ oscillations, Ca^2+^ ATPases of the ER were blocked by the administration of 2 µM thapsigargin. The transient rates of the slow Ca^2+^ oscillations were significantly reduced by the application of thapsigargin in both putative-neurons and astrocytes (Figure 4 C, E, F; transient rate relative to the control: 11±8% (n = 29 putative-neurons, 3 slices, 3 mice; p<0.0001) and 34±8% (n = 9 astrocytes, 2 slices, 2 mice; p<0.0001); one-sample t-test). Baselines of [Ca^2+^]_i_ were elevated by blockade of Ca^2+^ uptake into ERs after administration of thapsigargin. This result suggested that the Ca^2+^ release from the ER was main origin of the slow Ca^2+^ oscillation. The blocker of the IP_3_ receptor, 2-APB (100 µM), also reduced the transient rate in both types of cells (Figure 4D, E, F; transient rate relative to the control: 24±11% (n = 27 putative-neurons, 4 slices, 4 mice; p<0.0001) and 1±1% (n = 9 astrocytes, 3 slices, 3 mice; p<0.0001); one-sample t-test). These results suggest that the IP_3_-induced Ca^2+^ release from the intracellular Ca^2+^ store (ER) is the main origin of the slow Ca^2+^ oscillations.

### mGluR5 contributes to the slow Ca^2+^ oscillations in putative-neurons and astrocytes

**Figure 5:**
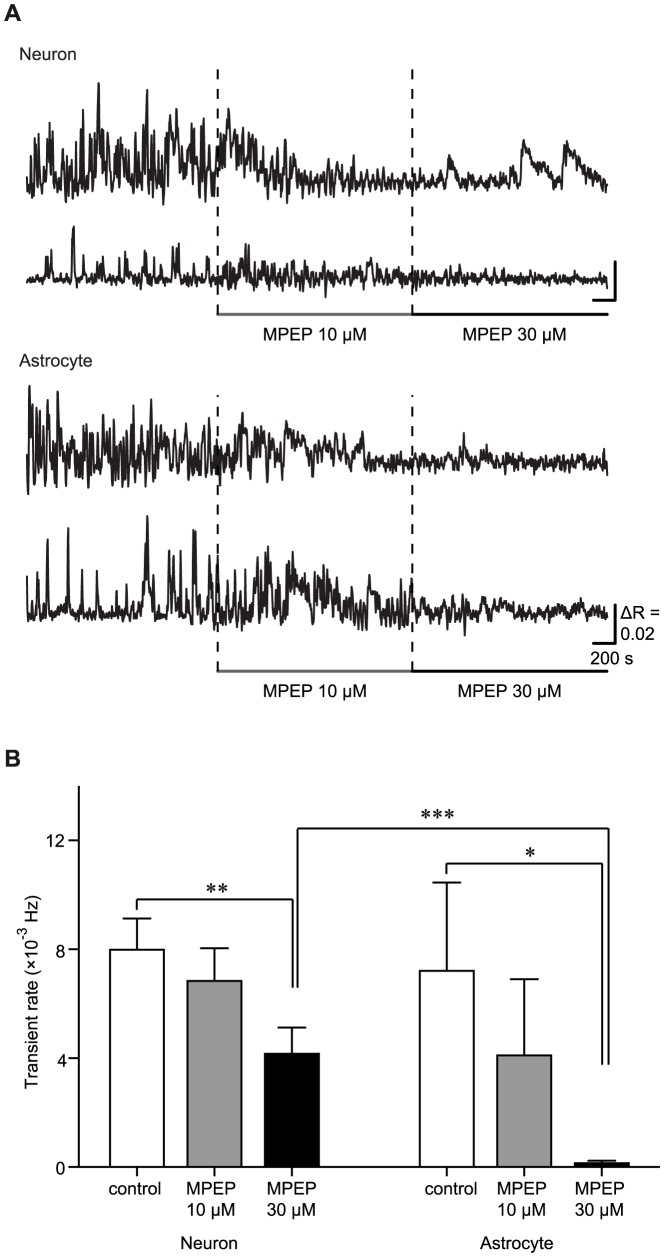
The slow Ca^2+^ oscillations in putative-neurons and astrocytes depend on mGluR5 activation. A, Typical time courses of the slow Ca^2+^ oscillations during the administration of MPEP in putative-neurons and astrocytes. Horizontal bars under the time courses indicate the period of MPEP application. Scale bar, 200 s, ΔR  = 0.02. B, Transient rates of the slow Ca^2+^ oscillations under the control condition and during the administration of 10 and 30 µM MPEP. The number of cells recorded is 30 putative-neurons and 8 astrocytes. *p<0.05; **p<0.03; ***p<0.01; Kruskal-Wallis test with Steel-Dwass post-hoc test.

mGluR5 is one of the receptors contributing to IP_3_ production and is abundant in the striatum [11]–[13]. Thus, we applied the mGluR5 blocker MPEP (Figure 5). 10 and 30 µM MPEP blocked the slow Ca^2+^ oscillations in a dose-dependent manner. When 30 µM MPEP was applied, the slow Ca^2+^ oscillations were almost completely blocked in astrocytes ((7.21±3.24) ×10^−3^ Hz for control and (0.07±0.05) ×10^−3^ Hz for 30 µM MPEP (n = 8 cells, 3 slices, 3 mice); p = 0.0295 vs. control; Kruskal-Wallis test with Steel-Dwass post-hoc test), and the transient rate of the slow Ca^2+^ oscillations in putative-neurons was significantly reduced ((7.98±1.15) ×10^−3^ Hz for control and (4.16±0.97) ×10^−3^ Hz for 30 µM MPEP administration (n = 30 cells, 3 slices, 3 mice); p = 0.0053 vs. control; Kruskal-Wallis test with Steel-Dwass post-hoc test). The effect of 30 µM MPEP on the slow Ca^2+^ oscillations in astrocytes was stronger than that in putative-neurons (p = 0.00389; Kruskal-Wallis test with Steel-Dwass post-hoc test). Average of the transient rates of the slow Ca^2+^ oscillations in the condition of 10 µM MPEP administration did not change significantly compared with that in the control condition. However, 10 µM MPEP changed the some properties of the Ca^2+^ oscillations (Figure S1). The low dose MPEP reduced the amplitude in putative-neurons significantly (Figure S1A; (1.54±0.96) ×10^−2^ under the control condition, n = 421 events, 30 cells, 3 slices, 3 mice; (1.48±0.70) ×10^−2^ with 10 µM MPEP administration, n = 316 events, 25 cells, 3 slices, 3 mice; mean ± SD; p = 0.0121; Kolmogorov-Smirnov test), but not in astrocytes. In the astrocytes, the decay slope was significantly decreased (Figure S1H; (1.69±1.26) ×10^−3^ under the control condition, n = 102 events, 8 cells, 3 slices, 3 mice; (7.39±1.07) ×10^−4^ with 10 µM MPEP administration, n = 42 events, 8 cells, 3 slices, 3 mice; mean ± SD; p = 0.0015; Kolmogorov-Smirnov test).

To confirm the contribution of mGluR1, another type of the group I mGluR, on the slow Ca^2+^ oscillations, LY367385, the specific antagonist of mGluR1, was applied. Figure S2 shows the effect of 50 µM LY367385 on the slow Ca^2+^ oscillations. The average values of the transient rates of the slow Ca^2+^ oscillations both in neurons and astrocytes did not alter with or without LY367385 significantly (Figure S2B; 26 putative-neurons and 9 astrocytes, 4 slices, 3 mice, p>0.05, Wilcoxon signed rank test). This result suggested that mGluR1 did not involve in the induction mechanisms of the slow Ca^2+^ oscillations.

### Neuronal activity regulates the slow Ca^2+^ oscillations in putative-neurons but not in astrocytes

**Figure 6:**
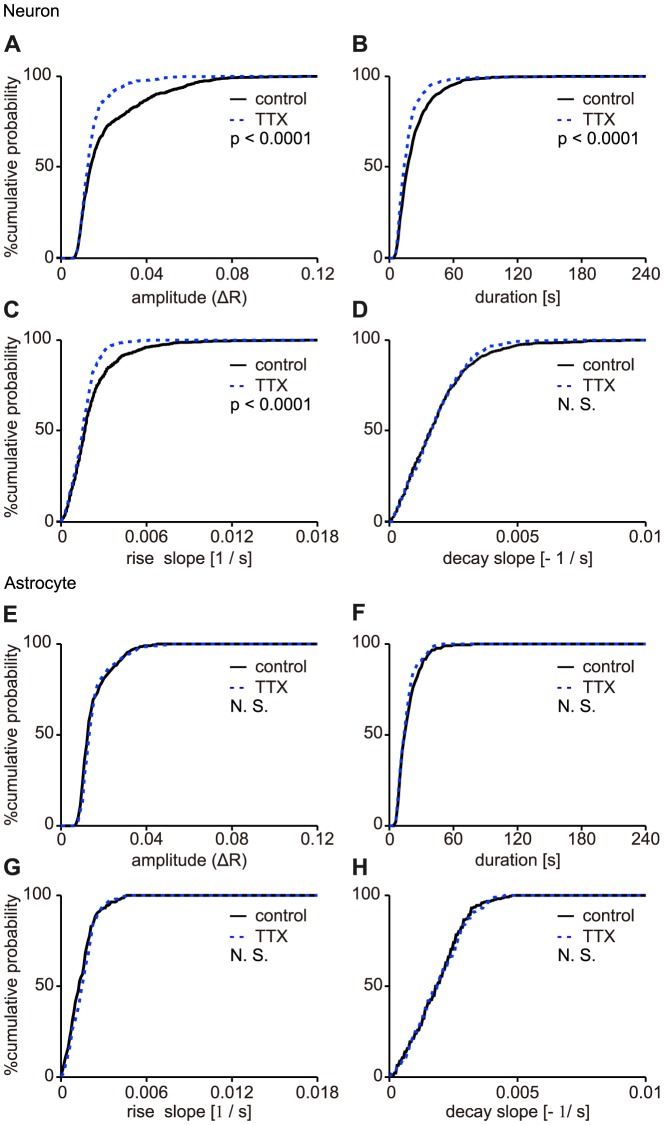
Changes in the properties of the slow Ca^2+^ oscillations in putative-neurons upon the blockade of action potentials. This figure shows the distribution of the peak amplitude (ΔR) (A, E), duration (B, F), rise slope (C, G), and decay slope (D, H) of the Ca^2+^ oscillations in cumulative probability plots for putative-neurons and astrocytes, respectively. The solid and dashed lines indicate the distribution of each parameter for the Ca^2+^ oscillations under the control condition and during the administration of TTX, respectively. *P*- values from the Kolmogorov-Smirnov test shown in the plots. N. S.: no significant difference.

To confirm the contribution of neuronal activity to the slow Ca^2+^ oscillations, the four parameters of the Ca^2+^ transients shown in Figure 3 for the 1 µM TTX condition were compared to those for the control condition. The distributions of those four parameters in putative-neurons and in astrocytes were shown in Figure 6A–D and 6E–H, respectively. The median values of the peak amplitude, duration, rise slope, and decay slope in putative-neurons were (1.39±1.66) ×10^−2^, 18±17 s, (1.67±1.82) ×10^−3^, and 1.60±1.37 ×10^−3^ under the control condition (n = 887 events, 62 cells, 7 slices, 6 mice, median ± SD), and (1.25±0.77) ×10^−2^, 14±15 s, (1.58±0.92) ×10^−3^, and 1.51±0.92×10^−3^ with TTX administration (n = 600 events, 44 cells, 7 slices, 6 mice,median ± SD), respectively. The distributions of the peak amplitude, duration, and rise slope of the Ca^2+^ transients in putative-neurons shifted toward smaller values under the TTX condition compared to the control condition (Figure 6A–C; p<0.0001, p<0.0001 and p<0.0001, respectively; Kolmogorov-Smirnov test). On the other hand, the distributions of the peak amplitude, duration, rise slope and decay slope of the Ca^2+^ transients in astrocytes did not change with TTX administration (Figure 6E–G; p = 0.0816, p = 0.5076, p = 0.1790, and p = 0.9520, respectively; Kolmogorov-Smirnov test). The median values of the peak amplitude, duration, rise slope, and decay slope in astrocytes were (1.23±0.74) ×10^−2^, 16±11 s, (1.30±0.96) ×10^−3^, and (1.85±1.02) ×10^−3^ under the control condition (n = 216 events, 24 cells, 4 slices, 4 mice, median ± SD) and (1.32±0.73) ×10^−2^, 14±9 s, (1.50±0.89) ×10^−3^, and (1.90±1.04) ×10^−3^ with TTX administration (n = 200 events, 20 cells, 4 slices, 4 mice, median ± SD), respectively. These results indicated that blockade of action potential reduced the amplitude, the duration and the rise slope of the slow Ca^2+^ oscillations in only putative-neurons but not in astrocytes.

**Figure 7:**
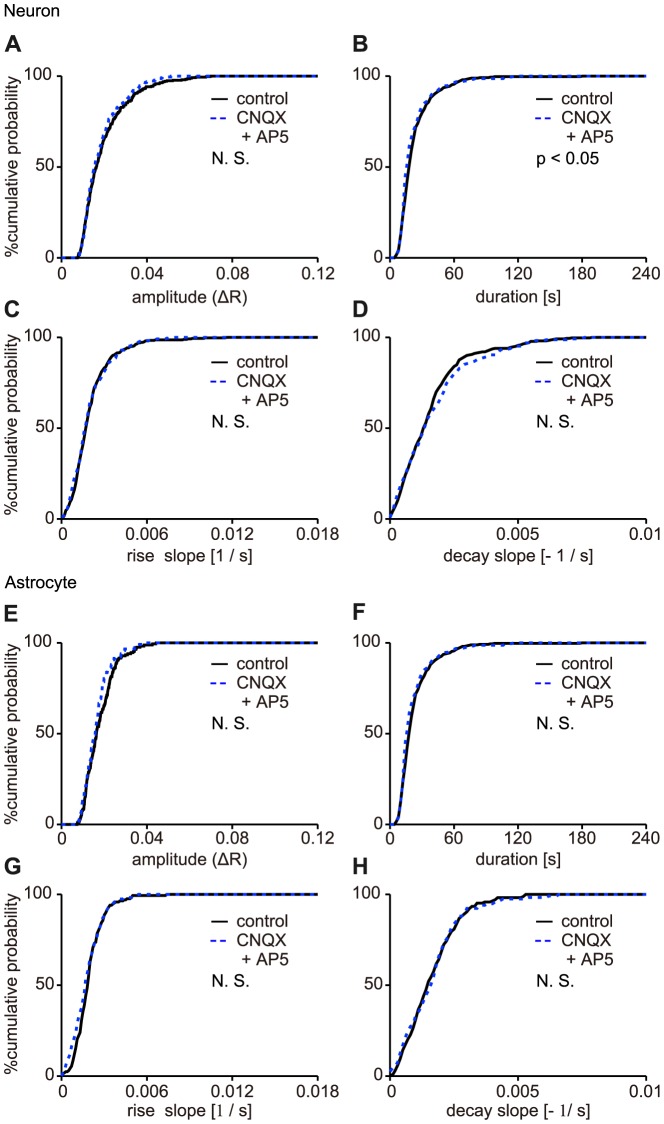
Changes in the properties of the Ca^2+^ oscillations in putative-neurons upon the blockade of ionotropic glutamate receptors. This figure shows the distribution of the peak amplitude (ΔR) (A, E), duration (B, F), rise slope (C, G), and decay slope (D, H) of the Ca^2+^ transients in cumulative probability plots for putative-neurons and astrocytes, respectively. The solid and dashed lines indicate the distribution of each parameter for the Ca^2+^ transients under the control condition and during the administration of CNQX and AP5, respectively. *P*- values from the Kolmogorov-Smirnov test shown in the plots. N. S.: no significant difference.

We also tested the contributions of the excitatory synaptic transmissions on the slow Ca^2+^ oscillations. Hence, antagonists of ionotropic glutamate receptors, 10 µM CNQX and 50 µM AP5, were applied. The distributions of those four parameters in putative-neurons and in astrocytes were shown in Figure 7A–D and 7E–H, respectively. The median values of the peak amplitude, duration, rise slope, and decay slope in putative-neurons were (1.64±1.12) ×10^−2^, 18±17.0 s, (1.75±1.51) ×10^−3^, and (1.35±1.36) ×10^−3^ under the control condition (n = 362 events, 20 cells, 4 slices, 4 mice, median ± SD) and (1.56±0.90) ×10^−2^, 16±17 s, (1.70±1.37) ×10^−3^, and (1.40±1.48) ×10^−3^ (n = 228 events, 18 cells, 4 slices, 4 mice, median ± SD) with CNQX and AP5 administration, respectively. And, the median values of those four parameters in astrocytes were (1.64±0.71) ×10^−2^, 18±9 s, (1.90±1.02) ×10^−3^, and 1.50±1.05×10^−3^ under the control condition (n = 167 events, 8 cells, 3 slices, 3 mice, median ± SD) and (1.58±0.62) ×10^−2^, 16±11 s, (1.70±1.05) ×10^−3^, and (1.60±1.19) ×10^−3^ with CNQX and AP5 administration (n = 117 events, 8 cells, 3 slices, 3 mice, median ± SD), respectively. The distributions of those four parameters of the Ca^2+^ transients, except for a slight difference in the duration in putative-neurons (Figure 7B; p = 0.0402; Kolmogorov-Smirnov test), were unchanged by the administration of CNQX and AP5 both in putative-neurons (Figure 7A, C, D; p = 0.7219, p = 0.5401, and p = 0.3253, respectively; Kolmogorov-Smirnov test) and astrocytes (Figure 7E–H; p = 0.0878, p = 0.3951, p = 0.3658, and p = 0.9942, respectively; Kolmogorov-Smirnov test). These results indicated that blockade of the excitatory synaptic transmission did not change all four parameters of the slow Ca^2+^ oscillations in both putative-neurons and astrocytes. These findings, in combination with the above results (Figure 5), suggest that neuronal activity may regulate the slow Ca^2+^ oscillations in putative-neurons but not in astrocytes through mechanisms other than action potentials inducing glutamatergic fast synaptic transmissions.

### Multicellular synchronicity of slow Ca^2+^ oscillations

**Figure 8:**
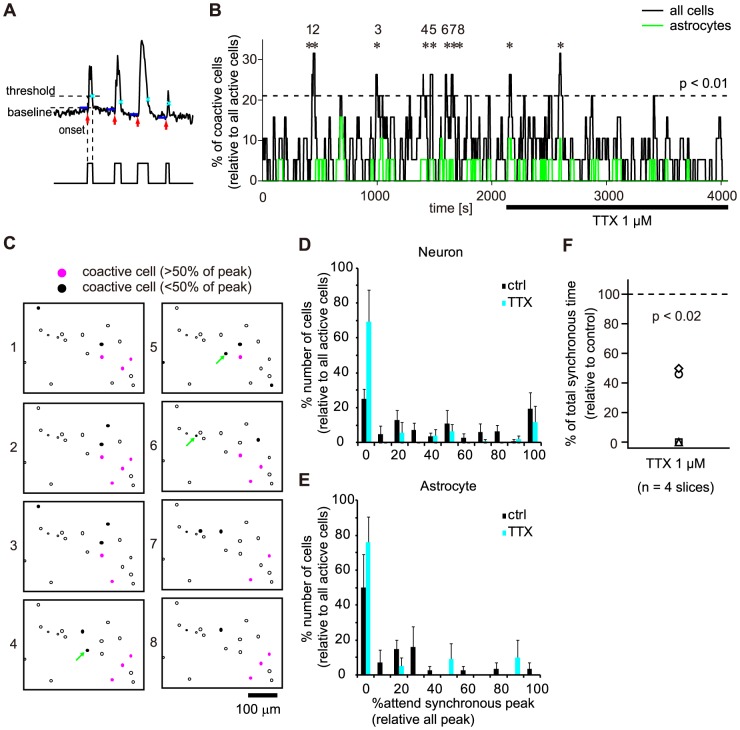
Neuronal activity-dependent multicellular synchrony of the Ca^2+^ oscillations. A, Schematic illustration for binarization of the Ca^2+^ oscillations. B, Histogram representing the percentage of co-active cells as a function of time. Asterisks (*) indicate significant synchronous peaks of spontaneous Ca^2+^ oscillations (see Materials and Methods). The black line indicates all cell types, and the green line indicates astrocytes only. The horizontal bar under the histogram indicates the period of TTX application. The dashed line represents a chance rate (p<0.01). C, Arrangement of active cells and co-active cells. Magenta and black filled circles indicate the cells active at more than and less than 50% of all peaks, respectively. Arrows indicate active astrocytes at each peak. Open circles indicate active cells that do not show activity at the peak. The numbers on the left side of each image correspond to the numbers of peaks shown in B. Scale bar, 100 µm. D, E, Histogram indicating the number of putative-neurons (D) and astrocytes (E) corresponding to peaks under the control (black bar) and TTX (cyan bar) conditions. F, Total synchronous time during the administration of TTX normalized to the control condition (n = 4 slices; p = 0.00706; one-sample t-test).

To elucidate the cell to cell correlations of the slow Ca^2+^ oscillations, we explored the simultaneity of the Ca^2+^ transients (Figure 8). Repeated synchronous activities of the slow Ca^2+^ oscillations over the chance rate were observed (Figure 8B). Figure 8C shows the active cells for each synchronous peak in an experiment. 35±6% of putative-neurons and 10±7% of astrocytes were active in more than 50% of synchronous peak (Figure 8C, magenta filled circles). This indicated that the a specific population of the putative-neurons and astrocytes (Figure 8C, green arrows) participated in the synchronous peak repeatedly. Figure 8D and 8E show the proportion of putative-neurons (D) and astrocytes (E) corresponding to peaks under the control (black bar) and 1 µM TTX (cyan bar) conditions. 75±6% of putative-neurons and 50±19% of astrocytes participated in synchronous peaks at least once under the control condition, and 31±18% of putative-neurons and 24±15% of astrocytes participated in synchronous peaks at least once under the TTX condition. The numbers of cells displaying synchrony in the slow Ca^2+^ oscillations decreased during TTX administration (Figure 8B, F; average of total synchronous time: 20±12% (%control); n = 4 slices, 4 mice; p = 0.0071; one-sample t-test). These results indicated that the slow Ca^2+^ oscillations in each cell were correlated by action potential-dependent mechanisms.

## Discussion

In this paper, we revealed that spontaneous slow Ca^2+^ oscillations, which involved long-lasting Ca^2+^ transients up to 200 s in duration, were exhibited in both astrocytes and putative-neurons (Figure 2). These Ca^2+^ oscillations were mainly due to the Ca^2+^ release from ER. This result show similar findings to our previous study in striatal cells of rats [17]. These slow Ca^2+^ oscillations in neurons resembles those reported for cultured neurons [9], [32], [33]. Tang and colleagues reported that the cultured striatal medium spiny projection neurons (MSNs) exhibited the slow Ca^2+^ oscillations under administration of dopamine or the agonists of dopamine receptors [9]. Yasumoto and colleagues showed that endogenous dopamine maintained synchronous oscillation of intracellular Ca^2+^ in primary cultured midbrain neurons [32]. But, the Ca^2+^ oscillations they found were blocked by administration of TTX. In other than mammals, cyclic AMP-dependent slow Ca^2+^ oscillation in *Xenopus* embryonic spinal cord neurons was reported by Gorbunova et al. [33]. The slow Ca^2+^ oscillations we found were spontaneous without any treatments, TTX-resistant, and not in cultured cells. Therefore, this is the first report of TTX-resistant slow Ca^2+^ oscillations in striatal putative-neurons in acute slice preparations without any activation, under cell-type discrimination enabled by the use of transgenic mice. The properties of the Ca^2+^ transients, such as the amplitudes, durations, rise slopes, and decay slopes, differed between putative-neurons and astrocytes (Figure 3). The slow Ca^2+^ oscillations exhibiting multicellular synchrony were observed (Figure 8) and were similar to action potential-induced [Ca^2+^]_i_ transients [3], [28], [34], in spite of TTX-resistant slow Ca^2+^ oscillations.

The possibilities that GFP-negative cells included the non-neuronal cells (see Results and Figure 1), and/or that GFP-negative non-neuronal cells (e.g. oligodendrocyte or microglia) may more frequently exhibit calcium events cannot be completely excluded. However, the possibility of no neuron expressing the slow Ca^2+^ oscillations should be low, because of following reasons. The properties of the individual Ca^2+^ transients in the control conditions and the effects of TTX on the properties of the Ca^2+^ transients were completely different between GFP-positive and –negative cells (Figure 3 and 6). This suggests that the both population of cells belonged to different groups. In addition, we confirmed that the slow Ca^2+^ oscillations were observed in striatal GABAergic neurons using GAD67-GFP knock-in mice [35] (unpublished observation). Thus, at least, it is safe to say that some neuron exhibit the slow Ca^2+^ oscillations, although slight possibility of the GFP-negative cells containing non-neuronal cells remains.

### Induction mechanisms of the slow Ca^2+^ oscillations

The slow Ca^2+^ oscillations in striatum were not induced by action potentials or glutamatergic fast synaptic transmissions (Figure 4A, B, E, F). The main source of the slow Ca^2+^ oscillations was Ca^2+^ release from the ER via IP_3_ receptors (Figure 4 C, D, E, F), and mGluR5-IP_3_ signaling pathways were associated with the generation mechanisms of the slow Ca^2+^ oscillations both in putative-neurons and astrocytes (Figure 5). However, the slow Ca^2+^ oscillations were partially blocked in putative-neurons under MPEP administration (Figure 5B). Thus, other receptors may be involved in the slow Ca^2+^ oscillations in putative-neurons. Indeed, striatal neurons express another type of mGluRs [11], [13], as well as dopamine receptors (DRs) [36], [37]. However, although we applied many antagonists against various type of receptors including mGluRs (MCPG, DL-AP3 and LY367385) and DRs (SCH23390 and Haloperidol), we could not find striking evidence for the contribution of another receptor (data not shown).

The administration of MPEP almost completely blocked the slow Ca^2+^ oscillations in astrocytes but not in putative-neurons (Figure 5B). There were two possibilities for accounting this phenomenon. The first possibility was that the activation of mGluR5 in astrocytes may induce some type of transmitter release from the astrocytes, which may then be received by the putative-neurons [38], [39]. mGluR5 was not only expressed at somatic region but also at processes of astrocytes [40]. Striatal neurons also expressed mGluR5 in both cell bodies and neurites [11], [41]. Thus, striatal neurons can communicate with astrocytes via mGluR5 throughout the cell. D'Ascenzo and colleagues reported that activation of mGluR5 induces Ca^2+^ oscillations in nucleus accumbens astrocytes with correlated appearance of NMDA receptor-dependent slow inward currents detected in MSNs [42]. This NMDA response in MSNs may generate Ca^2+^ oscillations. But, this is not the case, since blockade of the glutamate synaptic transmission with CNQX and AP5 did not affect on the slow Ca^2+^ oscillations in both putative-neurons and astrocytes. Astrocytes release adenosine triphosphate (ATP) in response to activation of mGluR5 and released ATP activates adenosine or purinergic receptors in neurons [38], [39], [43]. The adenosine or purinergic receptors can modulate Ca^2+^ signaling [44]–[46]. This process may have relevance to the Ca^2+^ oscillations in neurons. The second possibility was that some kind of metabotropic receptors other than mGluR5 concerned the Ca^2+^ oscillations in putative-neurons. However, as described above, we could not find striking evidence for the contribution of another receptors on the slow Ca^2+^ oscillations in the putative-neurons. Verification of the mechanisms of the neuronal slow Ca^2+^ oscillations will require further investigation.

Although TTX did not block the slow Ca^2+^ oscillations in both putative-neurons and astrocytes, cellular correlations of the Ca^2+^ elevation were reduced by the application of TTX (Figure 8B). mGluR5 was the main contributor of the slow Ca^2+^ oscillations. In the control condition, phasic or synchronous glutamate release from the cortical or thalamic efferents, leading to the multicellular synchronous Ca^2+^ oscillations. In the condition of TTX administration, the phasic glutamate release might not occur and the smaller amount of glutamate might be released compared with the control condition, thus the amplitudes of the individual transients of the Ca^2+^ oscillations (Figure 6A) and the occurrence of the multicellular synchrony of the Ca^2+^ oscillations (Figure 8B and F) might be reduced by TTX administration. Verification of this hypothesis will also require further investigation.

Sun and colleagues reported that expression of mGluR5 decreased in astrocytes of hippocampus and cortex developmentally [47]. They observed Ca^2+^ signals triggered by mGluR5 agonist in P12-15 mice. We also administered the mGluR5 antagonist to P11-17 mice. Thus, the older age of mice should be used for understanding the developmental change of the slow Ca^2+^ oscillations.

### Functional implications of the slow Ca^2+^ oscillations

Intracellular Ca^2+^ can modulate protein function, gene expression, and morphological changes in cellular processes [1]. Indeed, group I mGluR-mediated Ca^2+^ signaling contributes to the immediate early gene expression in cultured striatal neurons [8]. In general, group I mGluRs are able to initiate the Ca^2+^ transients, which may be critical for the gene expression-involved neuroplasticity important for physiological and pathophysiological changes in striatal functions [48]. Thus, the slow Ca^2+^ oscillations reported herein may relate to the gene expression.

Ca^2+^-activated K^+^ channels are also modulated by intracellular Ca^2+^. Two types of Ca^2+^-activated K^+^ channels, small conductance (SK) and large conductance (BK) channels, are expressed in the MSNs in striatum, and their currents represent between 30% and 50% of the sustained outward current [49], [50]. In globus pallidus neurons, SK channels influence voltage-gated ion channels to determine the precision of firing [51]. Clements et al. reported that IP_3_R-dependent Ca^2+^ release from intracellular Ca^2+^ stores suppressed MSN firing via Ca^2+^-activated K^+^ channels [52]. Our preliminary simulation study showed that these slow Ca^2+^ oscillations may affect the firing rate via Ca^2+^-activated K^+^ channels [53]. Thus, the slow Ca^2+^ oscillations reported herein may modulate the firing properties of MSNs on an intermediate time scale, one that is longer than an action potential and shorter than a circadian rhythm.

Both putative-neurons and astrocytes participate in the synchronous activities of the slow Ca^2+^ oscillations (Figure 8D–F). The neuron-glia interaction mediated by mGluR has been previously reported in several brain regions [2], [38], [39], [47]. Thus, it is possible that the slow Ca^2+^ oscillations concerning mGluR5 are one of the mediators of that neuron-glia interaction in the striatum.

In conclusion, we found the long-lasting slow Ca^2+^ oscillations in both putative-neurons and astorcytes. These slow Ca^2+^ oscillations were TTX-resistant and mGluR5-dependent. The slow Ca^2+^ oscillations exhibiting multicellular synchrony including both neurons and astrocytes were observed. This phenomenon was similar to the action potential-induced [Ca^2+^]_i_ transients [3], [28], [34], in spite of TTX-resistant slow Ca^2+^ oscillations. Intracellular Ca^2+^ can modulate the functions of various proteins, thus, the mGluR5-dependent slow Ca^2+^ oscillations we found may regulate the cellular functions leading to change the state of cellular networks in the striatum.

## Supporting Information

Figure S1 **The effect of the low-dose MPEP treatment on the properties of the Ca^2+^ oscillations.** This figure shows the distribution of the peak amplitude (ΔR) (A, E), duration (B, F), rise slope (C, G), and decay slope (D, H) of the Ca2+ transients in cumulative probability plots for putative-neurons and astrocytes, respectively. The solid and dashed lines indicate the distribution of each parameter for the Ca^2+^ transients under the control condition and during the administration of 10 µM MPEP, respectively. P- values from the Kolmogorov-Smirnov test shown in the plots. N. S.: no significant difference.
(PDF)

Figure S2 **Blocking mGluR1 did not alter the transient rate of the slow Ca^2+^ oscillations.** A, Typical time courses of the slow Ca^2+^ oscillations during the administration of 50 µM LY367385 in putative-neurons and astrocytes. Horizontal bars under the time courses indicate the application period of the agents. Scale bar, 200 s, ΔR  = 0.02. B, Transient rates of the slow Ca^2+^ oscillations during the administration of 50 µM LY367385 in putative-neurons and astrocytes. The number of cells recorded is 26 putative-neurons and 9 astrocytes (4 slices, 3 mice). The average values of the transient rates of the slow Ca^2+^ oscillations in putative-neurons were (7.59±0.95)×10^−3^ Hz under the control condition, and (6.94±0.76) ×10^−3^ Hz with LY367385 administration. The average values of the transient rates of the slow Ca^2+^ oscillations in astrocytes were (2.84±0.68) ×10^−3^ Hz under the control condition, and (3.61±0.53) ×10^−3^ Hz with LY367385 administration. The average values of the transient rates of the slow Ca^2+^ oscillations both in neurons and astrocytes did not alter with or without LY367385 significantly (p>0.05, Wilcoxon signed rank -test). N. S.: no significant difference.
(PDF)
