# Supplementary figures and images for: Both Neurons and Astrocytes Exhibited Tetrodotoxin-Resistant Metabotropic Glutamate Receptor-Dependent Spontaneous Slow Ca2+ Oscillations in Striatum
Source: PLoS One. 2014 Jan 15;9(1):e85351. doi: 10.1371/journal.pone.0085351 (PMC3893197; doi:10.1371/journal.pone.0085351)

Neuron

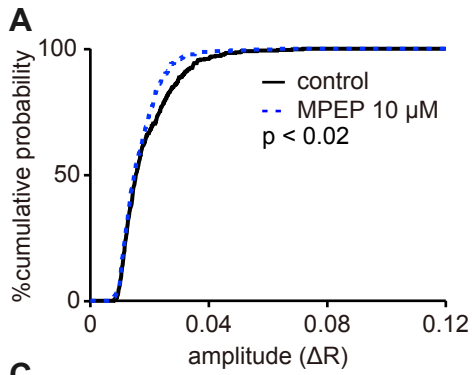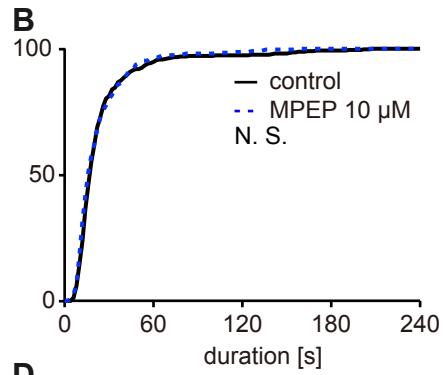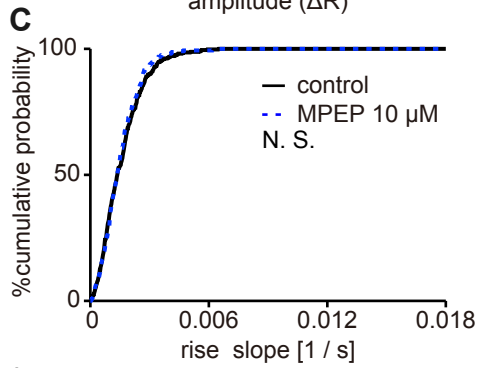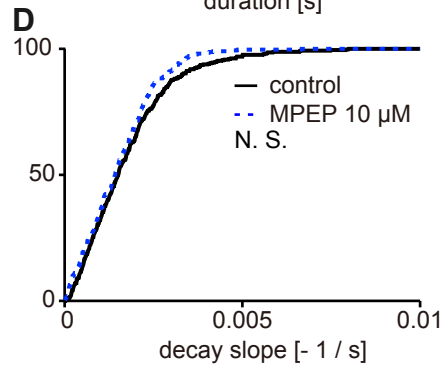

Astrocyte

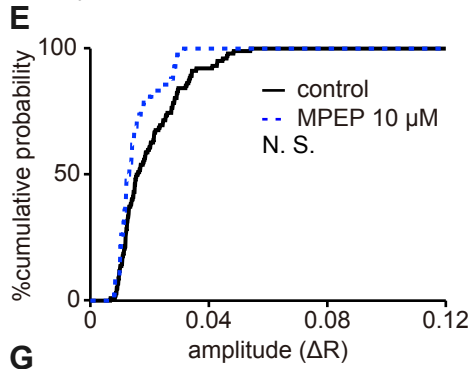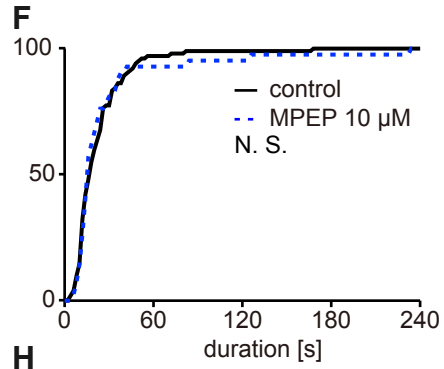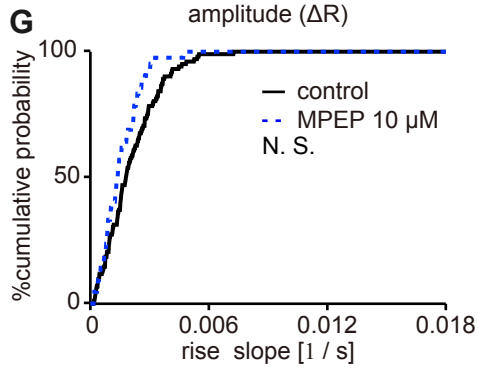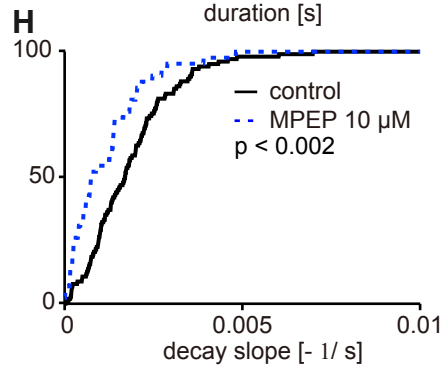

Supplement: Figure S1 — The effect of the low-dose MPEP treatment on the properties of the Ca2+ oscillations. This figure shows the distribution of the peak amplitude (ΔR) (A, E), duration (B, F), rise slope (C, G), and decay slope (D, H) of the Ca2+ transients in cumulative probability plots for putative-neurons and astrocytes, respectively. The solid and dashed lines indicate the distribution of each parameter for the Ca2+ transients under the control condition and during the administration of 10 µM MPEP, respectively. P- values from the Kolmogorov-Smirnov test shown in the plots. N. S.: no significant difference. (PDF) [file pone.0085351.s001.pdf]

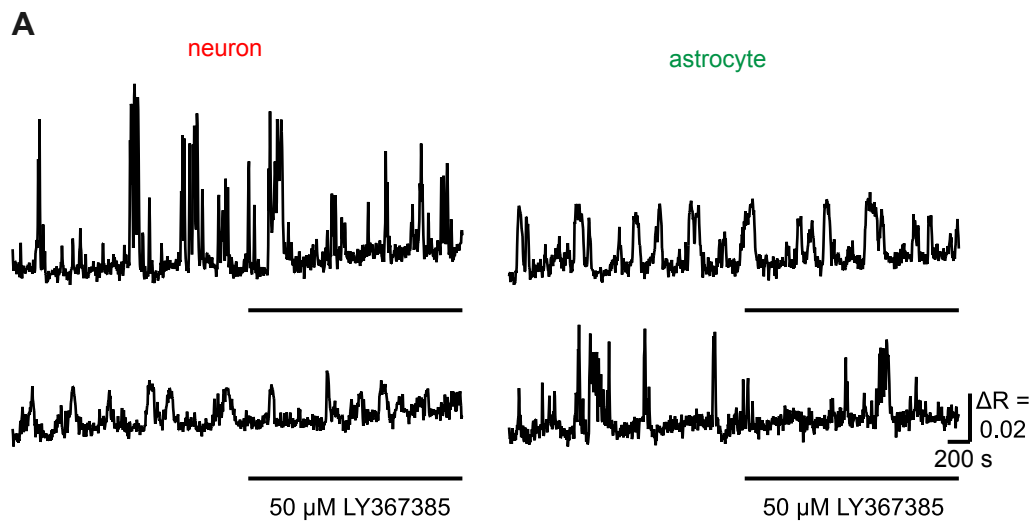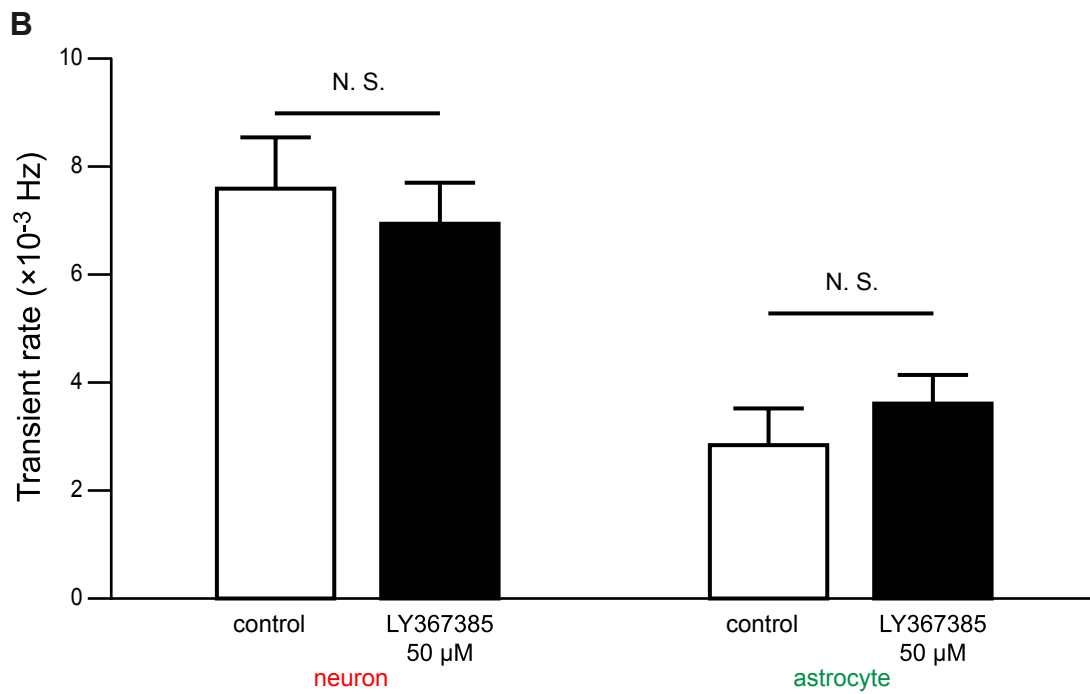

Supplement: Figure S2 — Blocking mGluR1 did not alter the transient rate of the slow Ca2+ oscillations. A, Typical time courses of the slow Ca2+ oscillations during the administration of 50 µM LY367385 in putative-neurons and astrocytes. Horizontal bars under the time courses indicate the application period of the agents. Scale bar, 200 s, ΔR = 0.02. B, Transient rates of the slow Ca2+ oscillations during the administration of 50 µM LY367385 in putative-neurons and astrocytes. The number of cells recorded is 26 putative-neurons and 9 astrocytes (4 slices, 3 mice). The average values of the transient rates of the slow Ca2+ oscillations in putative-neurons were (7.59±0.95)×10−3 Hz under the control condition, and (6.94±0.76) ×10−3 Hz with LY367385 administration. The average values of the transient rates of the slow Ca2+ oscillations in astrocytes were (2.84±0.68) ×10−3 Hz under the control condition, and (3.61±0.53) ×10−3 Hz with LY367385 administration. The average values of the transient rates of the slow Ca2+ oscillations both in neurons and astrocytes did not alter with or without LY367385 significantly (p>0.05, Wilcoxon signed rank -test). N. S.: no significant difference. (PDF) [file pone.0085351.s002.pdf]
